# Youth Kicker's Knee: Lateral Distal Femoral Hemiphyseal Arrest Secondary to Chronic Repetitive Microtrauma

**DOI:** 10.5435/JAAOSGlobal-D-19-00079

**Published:** 2019-08-06

**Authors:** Michael Dempewolf, Kevin Kwan, Benjamin Sherman, John A. Schlechter

**Affiliations:** From the Riverside University Health System, Moreno Valley, CA (Dr. Dempewolf, Dr. Sherman, and Dr. Schlechter); Western University of Health Sciences, Pomona, CA (Mr. Kwan); and Children’s Hospital of Orange County, Orange, CA (Dr. Schlechter).

## Abstract

Year-round competitive sports place the youth athlete at risk for injury from chronic repetitive stress. Stress injuries to the distal femoral physis in adolescents are rare. This report highlights three male youth soccer players who presented with a lateral distal femoral hemiphyseal arrest and a subsequent unilateral genu valgum deformity in their dominant “kicking leg” due to repetitive microtrauma, a phenomenon we refer to as youth kicker’s knee. Mean age was 14.2 years, and all participated in year-round soccer and American football. Imaging demonstrated aberration of the distal lateral femoral physis. All patients were surgically treated. Our series illustrates a unique presentation of a chronic overuse injury in hyper sporting adolescents resulting in an ipsilateral genu valgum deformity. Understanding adolescent growth and developmental characteristics is paramount to appropriate care, prevention, and treatment of physeal injuries that may occur from repetitive overuse and avoid surgery in these young athletes when possible.

Up to 50% of all injuries in pediatric sports medicine are related to overuse, most of which can be treated nonsurgically with little long-term sequelae.^1^ Apophyseal and physeal damage can occur secondary to acute or chronic injuries during youth sports participation. Cellular changes in the growing physis can result in growth irregularities, and when unrecognized, can lead to significant long-term deformity and disability.^2^ The purpose of this study is to report on a series of adolescent kickers treated for lateral distal femoral hemiphyseal arrest secondary to chronic repetitive microtrauma.

## Case Reports

This study was exempted by the institutional review board. A retrospective review of adolescents who presented to the orthopedic clinic for lateral distal femoral hemiphyseal arrest secondary to chronic repetitive kicking was performed for the period 2010 to 2018. History, physical examination, and imaging findings were obtained as well as the treatment course for all adolescents.

### History

All adolescents presented with right knee pain isolated to the medial aspect of the knee. All participated in competitive year-round soccer leagues, and one patient also participated in American football as a place kicker. All were right leg dominant, which was their “kicking leg.” Pain was activity related and mild/moderate in severity. Patient characteristics and symptoms are summarized in Table 1.

**Table 1:**
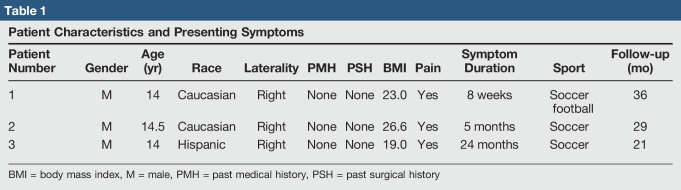
Patient Characteristics and Presenting Symptoms

### Physical Examination

All patients demonstrated full painless passive and active range of motion. Visual inspection of standing alignment was significant for unilateral right-sided genu valgum in all cases. Q angles ranged from 19° to 24° on the right and 8° to 14° on the left. Standard ligamentous stability testing was significant for 1+ laxity with valgus stress at 30° of knee flexion. Palpation of all standard knee anatomic landmarks was negative except in patient 2 who had tenderness over the medial knee with palpation.

### Radiographic Imaging

**Figure 1:**
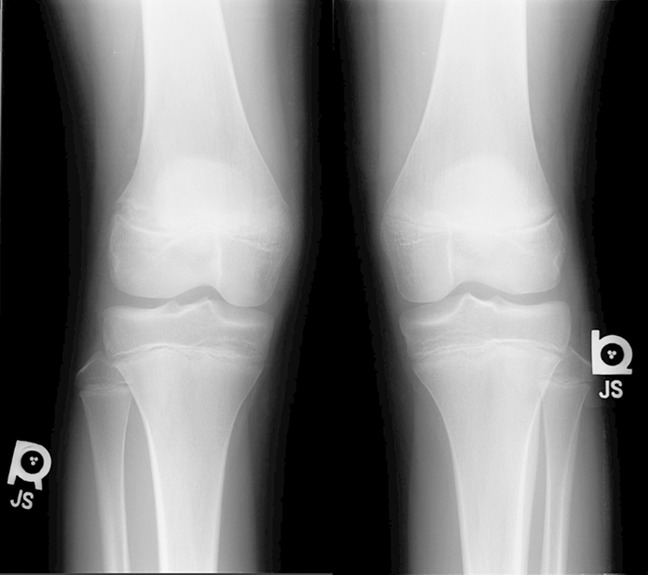
Radiographs of an adolescent boy with atraumatic lateral distal femoral physeal arrest. The right side (affected side) demonstrates physeal widening, while the left side (nonaffected side) demonstrates a normal appearing physis.

Standing alignment radiographs, four views of the right knee (AP, lateral, notch, and merchant), and left-hand x-rays (to determine bone age) were taken for all patients. Standing alignment radiographs demonstrated a mechanical axis deviation of + 2 right and neutral alignment left.^3^ The mechanical lateral distal femoral angle was measured on standing alignment radiographs and ranged from 78° to 82° right and 85° to 89° left. The lateral femoral physis was widened and irregular on radiographs (Figure 1). Physical examination and radiographic measurements are summarized in Table 2.

**Table 2:**
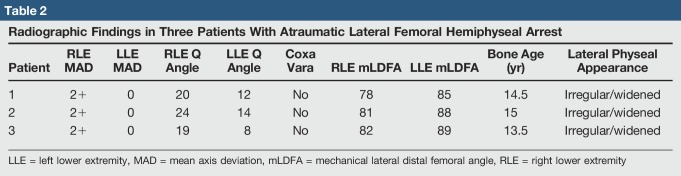
Radiographic Findings in Three Patients With Atraumatic Lateral Femoral Hemiphyseal Arrest

### Advanced Imaging

**Figure 2:**
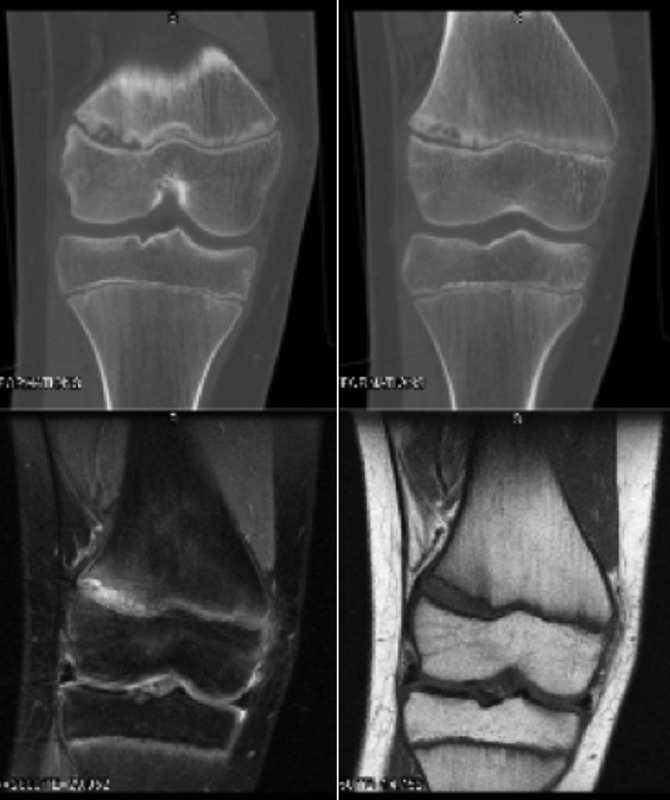
(**A**–**D**). CT and MRI sequences in an adolescent boy with atraumatic lateral distal femoral physeal arrest. CT demonstrates widening and fragmentation of the lateral physis (**A**, **B**). T2-weighted MRI sequences demonstrate increased signal and widening in the lateral distal femoral physis (**C**). T1-weighted images demonstrate physeal widening (**D**).

One adolescent (PN 2) had a right knee MRI and CT scan before to presentation in the orthopedic clinic. The CT and MRI series are depicted in Figure 2.

### Treatment

**Figure 3:**
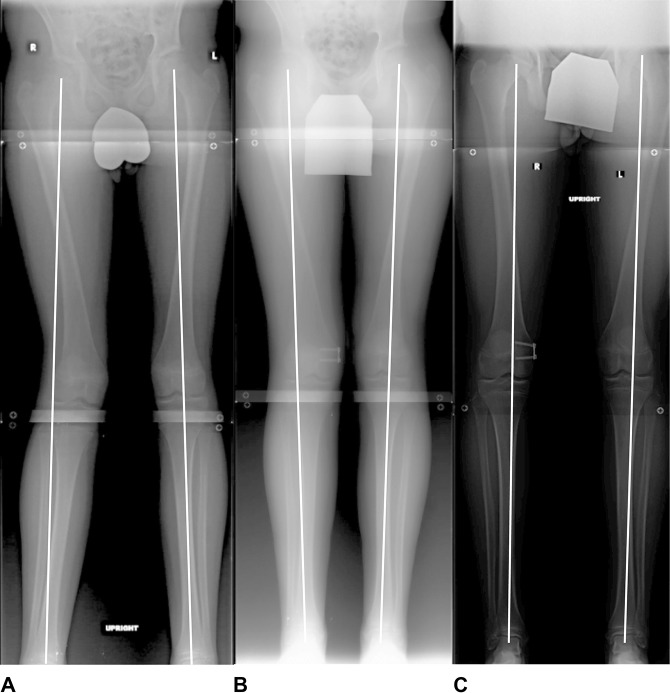
Serial standing alignment radiographs of an adolescent boy (patient #1) with lateral distal femoral hemiphyseal arrest treated with guided growth until resolution of his mechanical axis. **A**, demonstrates the patient’s presenting radiographs. **B**, represents the patient’s radiographs 4 months after starting guided growth with a medial tension band. **C**, represents the patient 15 months after initiation of guided growth with restoration of his mechanical axis.

**Figure 4:**
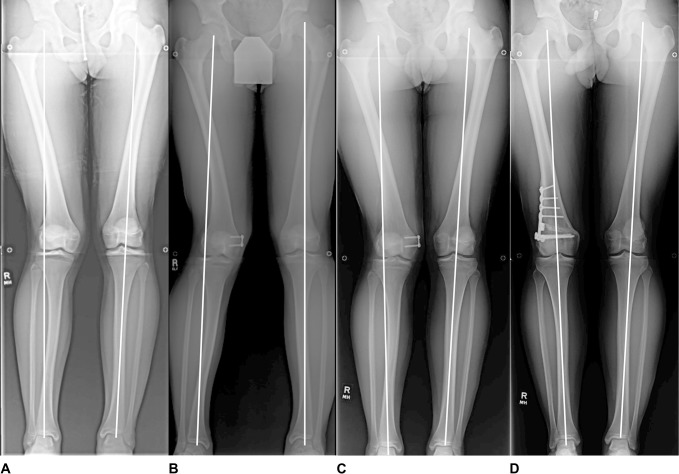
Serial standing alignment radiographs of an adolescent boy (patient #2) with lateral distal femoral hemiphyseal arrest treated unsuccessfully with guided growth and requiring a distal femoral osteotomy to realign his mechanical axis. **A**, demonstrates the patient’s presenting radiographs. **B**, represents the patient’s radiographs 6 months after starting guided growth with a medial tension band. **C**, represents the patient 9 months after initiation of guided growth with no improvement in his mechanical axis. **D**, represents the patient 6 months after undergoing a lateral opening wedge osteotomy with resolution in his mechanical axis.

**Figure 5:**
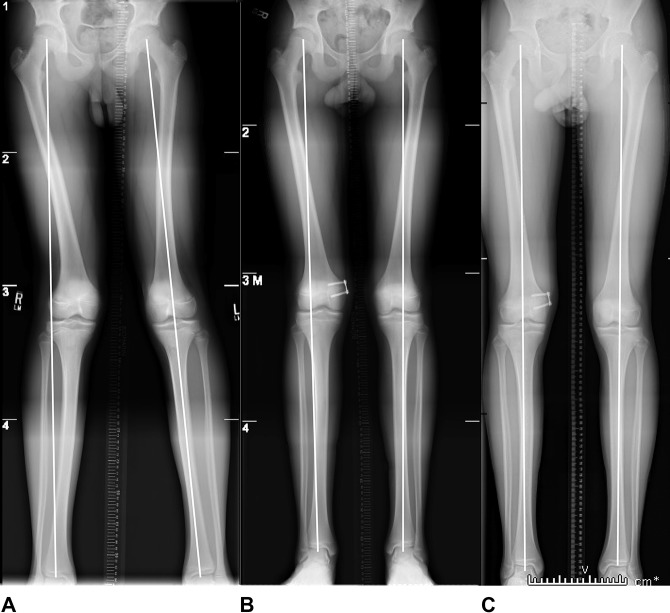
Serial standing alignment radiographs of an adolescent boy (patient #3) with lateral distal femoral hemiphyseal arrest treated with guided growth until resolution of his mechanical axis. **A**, demonstrates the patient's presenting radiographs. **B**, represents the patient’s radiographs 9 months after starting guided growth with a medial tension band. **C**, represents the patient 14 months after initiation of guided growth with restoration of his mechanical axis.

All patients underwent primary surgical correction via a medial hemiepiphysiodesis with insertion of a medial tension band plate for guided growth and gradual correction of the deformity. Serial examinations and standing alignment films were performed post-surgery. Radiographs were re-evaluated until restoration of a normal mechanical axis. Two patients responded well to guided growth with restoration of their mechanical axis (PN1,3). One limb malalignment failed to correct and the patient underwent removal of the medial tension band plate and a lateral opening distal femoral osteotomy (PN 2). The authors postulate that the reason for the failure of guided growth in PN2 was his more advanced bone age (15 years) compared with PN1 (14.5 years) and PN3 (13.5 years) on left-hand radiographs (Table 2). However, at that time, the senior author discussed treatment options with the family with the knowledge that the patient may not have enough growth remaining and may need an osteotomy in the future if the less invasive option (guided growth) failed. All adolescents returned to competitive soccer. Presenting and follow-up standing alignment radiographs for all patients are depicted in Figures 3–5.

## Discussion

The distal femoral physis serves as the primary location of endochondral ossification and provides the largest contribution to longitudinal growth of the lower extremity.^4^ Repetitive microtrauma can lead to disruption of the metaphyseal vasculature that supplies the physis with necessary substrates to promote normal endochondral ossification.^5^ Without appropriate perfusion, further ossification is halted, causing chondrocytes to accumulate in the proliferative zone and form long columns of hypertrophic cartilage.^5^ Radiographically, this can be seen as focal widening of the physis. This aberrancy is often temporary; however, if there is significant ischemia, osseous necrosis may ensue, leading to permanent growth disturbances as highlighted in this case series.^6^

There have been few cases of distal femoral physeal injuries in the literature, typically from trauma or resulting from fracture. A case report by Holloway et al.^7^ described a similar injury pattern in a 16-year-old soccer player who developed an asymmetric genu valgum deformity from a Salter-Harris V injury to the lateral distal femoral physis after an medial collateral ligament (MCL) injury. The patient was ultimately treated successfully using a distal femoral osteotomy.^7^ To our knowledge, this is the first report of a series of adolescents with lateral distal femoral hemiepiphyseal growth arrest leading to a significant limb deformity with the need for subsequent surgical correction.

The diagnosis of lateral distal femoral hemiphyseal arrest is complex because many other conditions may lead to a similar deformity or symptoms. Genu valgum deformities in children may be caused by congenital hip conditions such as coxa vara, acute trauma to the lateral femoral physis, increased body mass index, and metabolic conditions, none of which were present in our patients.^8,9,10,11^ In our study, the key diagnostic imaging finding was the appearance of a widened irregular physis. Laor et al.^12^ detailed the imaging findings of hemiepiphyseal widening of the distal femoral physis on MRI in six children who all participated in competitive sports. Interestingly, in their study, only one child presented with a deformity and none required surgical intervention.

Our study further demonstrates that overuse injuries in the growing child can cause significant long-term problems. We believe that the mechanism for lateral distal femoral growth arrest stems from repetitive valgus stress due to kicking a soccer ball or football, causing repetitive compression and damaging of the lateral physis. It is imperative that sports physicians, parents, coaches, and players be aware of the risk factors and signs of overuse to prevent chronic physeal injuries. These signs include both intrinsic and extrinsic factors that increase the risks as well as the potential for long-term consequences of injury. Intrinsic factors include age, body mass index, gender, and anatomic variations. Extrinsic factors reflect the type of athlete, and duration and intensity of training.^13^ These extrinsic factors should be addressed when assessing patients with suspected overuse injuries as well as those presenting for preparticipation physicals. Rapid increase in training load after long periods of inactivity, athletes performing at levels higher than their level of training, and those with consistent high-level sport activity (eg, those participating in sports year-round) with or without the presence of a professional coach are particularly prone to overuse injuries.^14^ The patients described in this report were at risk given their year-round participation in sports, high-intensity training, and skeletal immaturity.

In summary, chronic overuse injuries are an incredibly common sports injury, and in the skeletally immature, can lead to permanent growth disturbances that may necessitate surgical intervention. Overuse injuries can be avoided by modifying factors that put the young athlete at risk and intervene before the onset of permanent sequelae.
